# A study on pulmonary function in children with obstructive sleep apnea hypopnea syndrome

**DOI:** 10.1007/s11325-024-03043-y

**Published:** 2024-05-08

**Authors:** Chun-qin Zeng, Hu-wei Yuan, Hao-cheng Wang, Hong Yang, Yi-shu Teng

**Affiliations:** 1 Department of Otorhinolaryngology, Shenzhen Children’s Hospital, Shenzhen, 518038 Guangdong China; 2 Department of Otorhinolaryngology, Shenzhen Children’s Hospital, China Medical University, Shenzhen, 518038 Guangdong China

**Keywords:** Obstructive Sleep Apnea Syndrome, Pulmonary Function, Sleep Breathing Disorder Index, Children

## Abstract

**Objective:**

To investigate the pulmonary function of children with obstructive sleep apnea syndrome.

**Methods:**

A total of 328 children aged 3 to 12 years old who were evaluated for a sleep disorder from January 2022 to June 2023 were selected as the observation group, classified into mild, moderate, and severe categories based on the apnea hypopnea index. The number of children with mild, moderate, and severe obstructive sleep apnea is 228, 62, and 28 respectively. Additionally, 126 healthy individuals aged 3 to 13 years old undergoing health examinations during the same period were selected as the control group. All subjects underwent sleep respiratory monitoring, pulmonary function tests, and impulse oscillometry. Comparative analysis was performed on pulmonary function indices (forced vital capacity, maximum ventilation, inspiratory capacity, total lung capacity, and inspiratory reserve volume), and respiratory impedance indices (resonant frequency, total respiratory impedance, viscous resistance at 5 Hz, 20 Hz, and 35 Hz). Pulmonary function indices were also compared among patients in the observation group with mild, moderate, and severe conditions.

**Results:**

In the observation group, the FVC pre% of patients decreased by 10.5 ± 5.99 compared to the control group. The MVV of the control group decreased by 28.10 ± 2.22 compared to patients in the observation group. The IC of the control group decreased by 0.68 ± 0.44 compared to patients in the observation group. The TLC of the control group decreased by 1.354 ± 0.51 compared to patients in the observation group. The ERV of the control group decreased by 0.53 ± 0.30 compared to patients in the observation group. Additionally, the Fres, Zrs, R5, R20, and R35 of the observation group were higher than those of the control group by 10.73 ± 0.18, 1.78 ± 0.24, 0.11 ± 0.17, 0.86 ± 0.13, and 0.02 ± 0.21, respectively. In sum, the pulmonary function indices of the observation group were significantly lower than those of the control group, while the respiratory impedance indices were higher (*P* < 0.05). Within the observation group, the pulmonary function indices of severe patients were lower than those of moderate and mild patients, and moderate patients had lower pulmonary function indices than mild patients (*P* < 0.05).

**Conclusion:**

The pulmonary function of children with obstructive sleep apnea syndrome is impaired and varies in severity. There are significant differences in pulmonary function, underscoring the importance of monitoring pulmonary function in these children for clinical assessment and treatment prognosis.

## Introduction

Children with Obstructive Sleep Apnea Hypopnea Syndrome (OSAHS) experience frequent partial or complete upper airway obstruction during sleep, leading to hypoventilation or respiratory pauses, resulting in hypoxemia and hypercapnia [1]. OSAHS in children is a prevalent but underdiagnosed condition [2]. According to the American Academy of Pediatrics, obstructive sleep apnea syndrome affects 1% to 5% of children in the general pediatric population [3, 4]. Clinical manifestations of childhood OSAHS are diverse, including snoring, breathing pauses, mouth breathing, and often accompanied by restless sleep, nocturia, night terrors, as well as behavioral and attention problems in children. OSAHS has a relatively high incidence in children, characterized by intermittent sleep-disordered breathing, which can impact a child's growth, craniofacial development, and various organ systems. Therefore, it warrants close attention from healthcare professionals and parents, aiming for early diagnosis and intervention, with an emphasis on a multidisciplinary, comprehensive approach to prevent adverse effects of childhood OSAHS.

Currently, there is limited research on the relationship between different degrees of OSAHS and pulmonary function [5]. This study aims to investigate the variations in pulmonary ventilation function and airway hyperreactivity (AHR) among OSAHS patients with different severity levels by conducting pulmonary function tests on confirmed OSAHS patients through polysomnography, comparing mild and moderate to severe patients.

## Material and methods

### General information

A total of 328 children diagnosed with Obstructive Sleep Apnea Hypopnea Syndrome (OSAHS), who were admitted to our hospital from January 2022 to June 2023, were selected as the observation group. Additionally, 126 healthy individuals undergoing health examinations during the same period were chosen as the control group. The observation group consisted of 168 females and 160 males, aged between 3 and 12 years, with a mean body mass index (BMI) of (16.5 ± 3.2) kg/m^2^, and a disease duration ranging from 6 months to 8 years, with an average of (3.72 ± 1.55) years. The control group included 66 females and 60 males, with an age range of 3 to 13 years and a mean BMI of (15.4 ± 4.1) kg/m^2^. There were no statistically significant differences in age (*t* = 0.210,* P* > 0.05) or BMI (*t* = 1.381, *P* > 0.05) between the two groups, demonstrating their comparability.

Using the Obstructive Apnea–Hypopnea Index (OAHI) as a criterion, patients in the observation group were categorized into three severity levels: mild, with 228 cases (OAHI 1–5 events/h); moderate, with 62 cases (OAHI 5–10 events/h); and severe, with 38 cases (OAHI > 10 events/h).

### Inclusion and exclusion criteria

#### Inclusion criteria

Patients who provided informed consent voluntarily and had a clear understanding of the study; observation group members who met the diagnostic criteria for Obstructive Sleep Apnea Hypopnea Syndrome (OSAHS) outlined in the "Chinese guideline for the diagnosis and treatment of childhood obstructive sleep apnea (2020)"[6].

#### Exclusion criteria

Patients with concomitant respiratory system disorders; significant dysfunction in vital organs such as the heart, liver, or kidneys; metabolic diseases; malignancies; cognitive impairments; contraindications for pulmonary function testing, including aortic dissection, severe hyperthyroidism, severe hypertension, epilepsy, pneumothorax, giant bullae in the lungs, tympanic membrane perforation, acute respiratory tract infections, and pregnant women, among others.

### Testing methods

All subjects underwent sleep respiratory monitoring, pulmonary function testing, and impulse oscillometry. Pulmonary function tests were conducted according to the ATS standards. The standard used to assess the severity of sleep-disordered breathing in this study is based on the diagnostic criteria from the Chinese Guidelines for the Diagnosis and Treatment of Childhood Obstructive Sleep Apnea–Hypopnea Syndrome (2020). According to these criteria, the OAHI (Obstructive Apnea Hypopnea Index) for normal children is < 1 events/h, 1–5 events/h for mild OSAS (Obstructive Sleep Apnea Syndrome) patients, 5–10 events/h for moderate OSAS, and > 10 events/h for severe OSAS.

#### Sleep respiratory monitoring examination

Before instructing the examination, patients were advised to abstain from coffee, tea, and other substances within the preceding 24 h. They were also instructed to refrain from taking any medications. The examination was conducted in a quiet and comfortable environment to ensure optimal sleep conditions. Multiple-channel sleep monitoring devices were used to continuously monitor sleep for 7 h, recording parameters such as chest and abdominal movements, airflow during oral and nasal breathing, electroencephalography (EEG), electrocardiography (ECG), and chin muscle electromyography (EMG). The collected data were stored using information technology.

#### Pulmonary function testing

Pulmonary function testing was conducted using a pulmonary function testing apparatus. Patients assumed a seated position, with their noses gently clamped shut using a nose clip, and a mouthpiece securely attached. They maintained an upright posture with a slight upward tilt of the head to open the airway. Supporting their cheeks with their hands and firmly biting onto the mouthpiece, the tongue was positioned below the mouthpiece, and the lips were sealed tightly around it to prevent any air leakage. After completing the aforementioned preparations, the testing procedure was initiated.

#### Pulse oscillation assessment

Pulse oscillation lung function assessment was conducted using the Pulse Oscillation Lung Function Analyzer (Master Screen, Germany, Jaeger). Patients were seated, and their noses were gently clamped shut while holding the mouthpiece in place. Their head was slightly inclined backward, with both hands supporting the cheeks on either side. They maintained steady breathing for 1 min to measure parameters such as resonance frequency (Fres), total respiratory impedance (Zrs), and viscous resistance at 5 Hz, 20 Hz, and 35 Hz (R5, R20, R35).

### Outcome measures

1. Comparison of pulmonary function indices between the two groups, including forced vital capacity percentage of predicted (FVCpre%), Maximum Voluntary Ventilation (MVV), Inspiratory Capacity (IC), Total Lung Capacity (TLC), and Expiratory Reserve Volume (ERV).
2. Comparison of pulmonary function indices (FVC, MVV, IC, TLC, ERV) among the mild, moderate, and severe patients in the observation group.
3. Comparison of respiratory impedance indices between the two groups, including resonance frequency (Fres), total respiratory impedance (Zrs), and viscous resistance at 5 Hz, 20 Hz, and 35 Hz (R5, R20, R35).

### Statistical analysis

Data were analyzed using SPSS 22.0 statistical software. Continuous variables are presented as (x ± s), and comparisons between groups were conducted using the t-test. Multiple-group comparisons were performed using one-way analysis of variance (ANOVA), with post hoc LSD-t tests for pairwise comparisons. A significance level of *P* < 0.05 was considered statistically significant.

## Results

### Comparison of pulmonary function indices between the two groups

In the observation group, FVC, MVV, IC, TLC, and ERV were all significantly lower than those in the control group (*P* < 0.05). Refer to Table 1 for details.

**Table 1:** Comparison of pulmonary function indices between the two groups (L, x ± s)

	The observation group(*n* = 328)	The control group (*n* = 126)	*t*	*P*
FVC pre%	93.21 ± 18.31	82.71 ± 12.32	18.781	< 0.001
MVV	51.21 ± 4.32	79.31 ± 6.54	28.781	< 0.001
IC	2.56 ± 0.43	3.24 ± 0.87	6.563	< 0.001
TLC	3.21 ± 0.32	4.564 ± 0.83	14.321	< 0.001
ERV	1.12 ± 0.45	1.65 ± 0.75	6.871	< 0.001

### Comparison of pulmonary function among patients with different severity levels in the observation group

Within the observation group, patients with severe OSAHS exhibited significantly lower values for FVC, MVV, IC, TLC, and ERV compared to patients with moderate and mild OSAHS. Patients with moderate OSAHS also demonstrated significantly lower values for FVC, MVV, IC, TLC, and ERV compared to those with mild OSAHS (*P* < 0.05). Refer to Table 2 for details.

**Table 2:** Comparison of pulmonary function among patients with different severity levels in the observation group (L, x ± s)

	Mild OAHI 1–5 (*n* = 228)	Moderate OAHI 5–10 (*n* = 62)	Severe OAHI > 10 (*n* = 38)	*F*	*P*
FVCpre%	95.21 ± 12.32	92.36 ± 13.64	92.76 ± 12.54	5.454	0.005
MVV	53.01 ± 4.56	51.23 ± 4.32	47.54 ± 5.43	8.321	< 0.001
IC	2.67 ± 0.85	2.56 ± 0.68	2.34 ± 0.43	3.321	0.015
TLC	3.42 ± 0.45	2.56 ± 0.32	2.23 ± 0.32	53.132	< 0.001
ERV	1.14 ± 0.66	1.12 ± 0.45	1.21 ± 0.23	3.167	0.044

### Comparison of respiratory impedance indices

In the observation group, the respiratory impedance indices, including Fres, Zrs, R5, R20, and R35, were significantly higher than those in the control group (*P* < 0.05). Please refer to Table 3 for details.

**Table 3:** Comparison of respiratory impedance indices (x ± s)

	The observation group (*n* = 328)	The control group (*n* = 126)	*t*	*P*
Fres	22.89 ± 2.56	12.16 ± 2.38	29.645	< 0.001
Zrs	53.01 ± 4.56	51.23 ± 4.32	8.675	< 0.001
R5	2.67 ± 0.85	2.56 ± 0.68	7.133	0.015
R20	3.42 ± 0.45	2.56 ± 0.32	8.965	< 0.001
R35	1.14 ± 0.66	1.12 ± 0.45	6.873	< 0.001

The results of this study demonstrate that in the observation group, values for FVC, MVV, IC TLC, and ERV were all lower than those in the control group, while indices of respiratory impedance, including Fres, Zrs, R5, R20, and R35, were significantly higher than those in the control group. Moreover, patients with severe OSAHS within the observation group exhibited greater reductions in FVC, MVV, IC, TLC, and ERV when compared to those with moderate and mild OSAHS, with the patients with moderate OSAHS also demonstrating more substantial reductions than those with mild OSAHS (*P* < 0.05). These findings indicate that pulmonary function indices are valuable for the assessment of OSAHS patients and can assist in distinguishing the severity of the condition. This is attributed to the fact that OSAHS patients experience respiratory disruptions during sleep, leading to damage to pulmonary and airway function. Severe cases result in prolonged periods of abnormal lung and airway function, which in turn exacerbates the degree of pulmonary damage, underscoring the importance of improving pulmonary and respiratory function during clinical management [7].

## Discussion

Obstructive Sleep Apnea Hypopnea Syndrome (OSAHS) is a condition with multiple influencing factors, primarily characterized by upper airway obstruction leading to airway narrowing, collapse, and resultant respiratory pauses. Clinical manifestations of OSAHS primarily include daytime somnolence, nocturnal snoring, and can further lead to decreased memory, weakened immune function, significantly impacting patients' work and quality of life [8]. Therefore, early diagnosis and timely treatment of OSAHS are crucial. Relevant studies have indicated that OSAHS has a significant impact on the respiratory system, with pulmonary function being an important assessment parameter for the respiratory system [7]. Conversely, chronic lung diseases (such as asthma, copd, interstitial lung disease, etc.) also increase the risk of later developing OSAHS. Therefore, pulmonary function examination is helpful to detect early lung function impairment in patients with OSAHS and potential patients with OSAHS, thereby providing theoretical support for clinical assessment and treatment prognosis. Regular monitoring of pulmonary function is essential for early diagnosis and treatment to reduce complications, as well as severe impairments such as fatalities and disabilities, ultimately alleviating the disease burden on the population and enhancing the quality of life while safeguarding public health [9].

Some researchers have suggested that children with enlarged tonsils and/or adenoids may exhibit altered pulmonary function [10, 11]. As early as 1997, foreign scholars [12] proposed the concept of "one airway, one disease," suggesting that disruptions in the upper and lower airways often coexist due to their continuous anatomical and histological features. On the other hand, upper airway obstruction can lead to changes in other parts of the respiratory system, affecting the activity of pharyngeal muscles and chest movement. During sleep, the autonomous and voluntary nervous systems controlling the upper airway do not coordinate effectively [13], and anatomical narrowing of the upper airway and abnormal pharyngeal muscle function promote the development of increased airway resistance. Airway obstruction can lead to mouth breathing, reduced airflow, increased respiratory effort, shortness of breath, and gas exchange disturbances, potentially resulting in hypoxia and hypercapnia [14]. Obstructive sleep apnea primarily occurs during the rapid eye movement (REM) sleep phase in children, during which neural activity that stimulates pharyngeal muscle activity diminishes. This results in hypoxemia and hypercapnia, reduced central nervous system responsiveness to CO2, decreased intercostal muscle activity, and reduced ventilation drive [15].

The impact of mouth breathing on the lower respiratory tract is thought to occur due to various mechanisms. In OSAHS patients, upper airway collapse and obstruction during sleep often lead to mouth breathing, which involves inhaling unfiltered, unheated, and unhumidified air into the lower airways, which can irritate the bronchi. Mouth breathing reduces the production of nasal-derived nitric oxide (NO), which plays a critical role in regulating nasal mucosal blood flow, antimicrobial and antiviral activity, and regulating airway resistance, gas exchange efficiency, and lung function [16]. Klinger et al. [17] found that patients with pulmonary hypertension have abnormal synthesis and signaling of NO in their blood vessels, resulting in higher pulmonary vascular resistance than in normal individuals after prolonged mouth breathing. Nasal breathing stimulates spontaneous ventilation due to nasal receptors, and switching from nasal to mouth breathing reduces this stimulation, leading to decreased ventilation and respiratory rates. Consequently, mouth breathing can lead to abnormal lung function and affects indicators of lung capacity [15].

Pulmonary function testing, as a non-invasive method, can aid in the early detection of the impact of pediatric Obstructive Sleep Apnea Hypopnea Syndrome (OSAHS) on the lower airway even in the absence of clinical or imaging evidence [13]. Therefore, pulmonary function testing plays an adjunctive diagnostic role in the early diagnosis and treatment of children with enlarged tonsils and/or adenoids, providing guidance for proactive surgical interventions. Timely improvements in nocturnal ventilation and the correction of nocturnal hypoxia can prevent further damage to small airway function. However, conducting pulmonary function tests in children is a challenging endeavor and often yields results that lack repeatability and high quality. This challenge is particularly evident in preschool-aged children who may not perform specific ventilatory maneuvers, requiring patience and experience from the testing personnel [18].

The impact of OSAHS on children's pulmonary function and the correlation between pulmonary function and the gold standard in sleep testing still lack large-scale clinical randomized controlled studies, indicating an area that requires further focused research. In conclusion, in future clinical practice, early comprehensive ear, nose, and throat examinations should be conducted for children with poor pulmonary function. This can help elevate the diagnostic and treatment proficiency of clinical practitioners and facilitate the control of lower airway research through the management of upper airway conditions, ultimately alleviating children's suffering, promoting their physical and mental development.

This study has found that differences in pulmonary function changes between the moderate-to-severe OSAHS group and the mild OSAHS group are statistically significant (*P* < 0.05). Patients with moderate-to-severe OSAHS exhibit more pronounced alterations in pulmonary ventilation function, which are directly related to the severity of OSAHS [8]. The study reveals that compared to patients in the mild OSAHS group, those with moderate-to-severe OSAHS exhibit a significantly higher proportion of Airway Hyperreactivity (AHR), with a statistically significant difference between the two groups. An increase in airway hyperresponsiveness is observed in patients with moderate-to-severe OSAHS, which may be due to the following factors:(1) Neuro reflex Mechanism: OSAHS patients experience increased vagal nerve tension during the night, leading to bronchoconstriction. Additionally, recurrent episodes of apnea stimulate receptors in the vocal cords and pharynx, causing reflex bronchoconstriction [19]. Furthermore, hypoxemia in OSAHS patients increases their reactivity to acetylcholine due to enhanced stimulation of the carotid body, leading to increased airway hyperresponsiveness. (2) Mechanical Stress on Airway Mucosa: When OSAHS patients experience apnea episodes and forcefully inhale, there is an increase in negative pressure within the chest, which causes some damage to the upper airway mucosa. This damage triggers an inflammatory response in the airway mucosa. Airway inflammation not only affects airway caliber and gas flow but also increases airway hyperresponsiveness [20]. Besides, numerous pathophysiologic links are implicated in OSAHS causing or worsening asthma [5]. Apneic episodes can increase cholinergic tone, activating muscarinic receptors in the airway leading to bronchoconstriction [21]. Apneas can increase thoracic blood volume, worsening spirometric indices of airflow obstruction [22]. Acute hypoxemia itself worsens bronchial reactivity [23]. These processes lead to airway remodeling and can favor the development of neutrophilic, difficult-to-treat asthma [24]. In clinical practice, it is advisable to test patients with poor PFT results for OSA and concurrently implement intervention to prevent further progression of their OSA which may lead to improvement in the underlying pulmonary disorder, i.e. asthma or fibrosis etc.

OSAHS is a condition that can lead to multi-organ system damage and has gained increasing attention from clinical practitioners and patients. OSAHS patients exhibit impaired pulmonary ventilation function and increased airway hyperresponsiveness, with a higher risk associated with higher AHI indices. This study suggests that regular monitoring of pulmonary ventilation function and timely testing of airway hyperresponsiveness should be conducted for OSAHS patients, aiming for early diagnosis and treatment to minimize the occurrence of complications.

### Limitations

This is one measurement in time. This could have been influenced by underlying asthma etc. which has yet to be diagnosed because the comorbid history is not known.

## Data Availability

The datasets used and/or analysed during the current study are available from the corresponding author (YS. T, tys118@163.com) on reasonable request.

## References

[1] Zhang W, Zhao ZR, Dai CF et al (2017) Correlation between Calpain-10 single-nucleotide polymorphisms and obstructive sleep apnea/ hypopnea syndrome with ischemic stroke in a Chinesepopulation: A population-based study. Medicine (Baltimore) 96:e6570 28422847 10.1097/MD.0000000000006570 PMC5406063

[2] Kadmon G, Shapiro CM, Chung SA et al (2013) Validation of a pediatric obstructive sleep apnea screening tool. Int J Pediatr Otorhinolaryngol 77:1461–1464 23838544 10.1016/j.ijporl.2013.06.009

[3] Marcus CL, Brooks LJ, Ward SD et al (2012) Diagnosis and management of childhood obstructive sleep apnea syndrome. Pediatrics 130:e714–e755 22926176 10.1542/peds.2012-1672

[4] Garg RK, Afifi AM, Garland CB et al (2017) Pediatric obstructive sleep apnea: consensus, controversy, and craniofacial considerations. Plast Reconstr Surg 140:987–997 29068938 10.1097/PRS.0000000000003752

[5] Alkhalil M, Schulman E, Getsy J (2009) Obstructive sleep apnea syndrome and asthma: what are the links? J Clin Sleep Med 5:71–78 19317386 10.5664/jcsm.27397 PMC2637171

[6] NI X (2020) Chinese guideline for the diagnosis and treatment of childhood obstructive sleep apnea (2020). Chin J Evid Based Med 20:883–900

[7] Chen RY, Ma XH, Sun T et al (2019) The effect of short-term, intensive rehabilitation exercises on the respiration, life quality and sleep of persons with obstructive sleep apnea and chronic obstructive pulmonary disease. Chin J Phys Med Rehabil 41:353–358

[8] Chen YJ, Zhao Y, Ai HJ et al (2017) Correlation of exercise cardiopulmonary function and severity and prognosis of obstructive sleep apnea-hypopnea syndrome. Anhui Med Pharm J 21:267–269

[9] Li Z, Xian JF, Ye JY (2018) Cine MRI evaluation on site and pattern of upper airway obstruction patients with obstructive sleep apnea-hypopnea syndrome during natural sleep. Chin J Med Imagining Techol 34:990–993

[10] Yadav SPS, Dodeja OP, Gupta KB et al (2003) Pulmonary function tests in children with adenotonsillar hypertrophy. Int J Pediatr Otorhinolaryngol 67:121–125 12623147 10.1016/S0165-5876(02)00351-8

[11] Fekri MS, Mianroodi AA, Shakeri H et al (2016) Effects of tonsil size on pulmonary function test results after tonsillectomy in children. Iran J Otorhinolaryngol 28:61 26878005 PMC4735618

[12] Caimmi D, Marseglia A, Pieri G et al (2012) Nose and lungs: one way, one disease. Ital J Pediatr 38:1–5 23098057 10.1186/1824-7288-38-60 PMC3507672

[13] Ehsan Z, Ishman SL (2016) Pediatric obstructive sleep apnea. Otolaryngol Clin North Am 49:1449–1464 27810015 10.1016/j.otc.2016.07.001

[14] Guan LJ, Zhang YM (2016) The effect of obstructive sleep apnea-hypopnea syndrome on pulmonary function of child: a literature review. J Otolaryngol Ophthal Shandong Univ 30:75–80

[15] DelRosso LM (2016) Epidemiology and diagnosis of pediatric obstructive sleep apnea. Curr Probl Pediatr Adolesc Health Care 46:2–6 26573241 10.1016/j.cppeds.2015.10.009

[16] Liu QH, Wu WJ (2004) Physiological and pathological effects of rhinogenic nitric oxide. Otolaryngol Foreign Med Sci 72–74

[17] Klinger JR, Abman SH, Gladwin MT (2013) Nitric oxide deficiency and endothelial dysfunction in pulmonary arterial hypertension. Am J Respir Crit Care Med 188:639–646 23822809 10.1164/rccm.201304-0686PP

[18] Xu J, Zhang YM, Xiang L (2014) The effect of obstructive sleep apnea-hypopnea syndrome on pulmonary function of child: a literature review. Chin J Pract Intern Med 34:45

[19] Zhang J, Zhou KP, Dai GY et al (2019) Effects of intraoperative and postoperative individualized nursing strategies on blood pressure, sleep and quality of life of patients with obstructive sleep apnea hypopnea syndrome. Hebei Med J 41(937–940):944

[20] Xu LM (2019) Observation on the Change of Pulmonary Function of Children with Obstructive Sleep Apnea Hypopnea Syndrome. World J Sleep Med 6:102–104

[21] Morrison JFJ, Pearson SB, Dean HG (1988) Parasympathetic nervous system in nocturnal asthma. BMJ 296:1427–1429 3132275 10.1136/bmj.296.6634.1427 PMC2545890

[22] DesJardin JA, Sutarik JM, Suh BY, Ballard RD (1995) Influence of sleep on pulmonary capillary volume in normal and asthmatic subjects. Am J Respir Crit Care Med 152:193–198 7599823 10.1164/ajrccm.152.1.7599823

[23] Dagg KD, Thomson LJ, Clayton RA, Ramsay SG, Thomson N (1997) Effect of acute alterations in inspired oxygen tension on methacholine induced bronchoconstriction in patients with asthma. Thorax 52:453–457 9176538 10.1136/thx.52.5.453 PMC1758550

[24] Taillé C, Rouvel-Tallec A, Stoica M, Danel C, Dehoux M, Marin-Esteban V, Pretolani M, Aubier M, D’Ortho M (2016) Obstructive Sleep Apnoea Modulates Airway Inflammation and Remodelling in Severe Asthma. PLoS ONE 11:e0150042 26934051 10.1371/journal.pone.0150042 PMC4774979

